# Preparation of Photocrosslinked Fish Elastin Polypeptide/Microfibrillated Cellulose Composite Gels with Elastic Properties for Biomaterial Applications

**DOI:** 10.3390/md13010338

**Published:** 2015-01-09

**Authors:** Shinya Yano, Megumi Mori, Naozumi Teramoto, Makoto Iisaka, Natsumi Suzuki, Masanari Noto, Yasuko Kaimoto, Masashi Kakimoto, Michio Yamada, Eri Shiratsuchi, Toshiaki Shimasaki, Mitsuhiro Shibata

**Affiliations:** 1 Department of Life and Environmental Sciences, Faculty of Engineering, Chiba Institute of Technology, 2-17-1 Tsudanuma, Narashino, Chiba 275-0016, Japan; E-Mails: shinya.y08232@gmail.com (S.Y.); megumi.m07232@gmail.com (M.M.); makoto.i10230@gmail.com (M.I.); natsumi.s09231@gmail.com (N.S.); masanari.n11231@gmail.com (M.N.); yasuko.k05230@gmail.com (Y.K.); masashi.k06230@gmail.com (M.K.); shimasaki.toshiaki@it-chiba.ac.jp (T.S.); mitsuhiro.shibata@p.chibakoudai.jp (M.S.); 2 Research & Development Division, Hayashikane Sangyo Co., Ltd., 2-4-8 Yamato-machi, Shimonoseki, Yamaguchi 750-8608, Japan; E-Mails: myamada@hayashikane.co.jp (M.Y.); esiratsuti@hayashikane.co.jp (E.S.)

**Keywords:** elastin, fish peptide, microfibrillated cellulose, hydrogel, photocrosslinking, composite gel

## Abstract

Photocrosslinked hydrogels reinforced by microfibrillated cellulose (MFC) were prepared from a methacrylate-functionalized fish elastin polypeptide and MFC dispersed in dimethylsulfoxide (DMSO). First, a water-soluble elastin peptide with a molecular weight of *ca.* 500 g/mol from the fish bulbus arteriosus was polymerized by *N*,*N*′-dicyclohexylcarbodiimide (DCC), a condensation reagent, and then modified with 2-isocyanatoethyl methacrylate (MOI) to yield a photocrosslinkable fish elastin polypeptide. The product was dissolved in DMSO and irradiated with UV light in the presence of a radical photoinitiator. We obtained hydrogels successfully by substitution of DMSO with water. The composite gel with MFC was prepared by UV irradiation of the photocrosslinkable elastin polypeptide mixed with dispersed MFC in DMSO, followed by substitution of DMSO with water. The tensile test of the composite gels revealed that the addition of MFC improved the tensile properties, and the shape of the stress–strain curve of the composite gel became more similar to the typical shape of an elastic material with an increase of MFC content. The rheology measurement showed that the elastic modulus of the composite gel increased with an increase of MFC content. The cell proliferation test on the composite gel showed no toxicity.

## 1. Introduction

Elastin is one of the most important proteins often seen in connective tissues of vertebrates. It has a unique molecular structure and contains many hydrophobic amino acids such as glycine, alanine, proline, and valine. Unlike collagen, elastin plays a role of a flexible component in tissues due to their unique structure [1,2]. In the process of elastin biosynthesis, first, a water soluble protein with a molecular weight of *ca.* 72 kDa—tropoelastin—is synthesized. Subsequently to the tropoelastin synthesis, crosslinking of multiple tropoelastin molecules with lysyl oxidase forms the insoluble protein elastin, and unique amino acids—desmosine and isodesmosine—are seen at the crosslinking points of elastin [1,3].

Recently, elastin and polypeptides with a repeating sequence derived from elastin have been attracting attention as biomaterials [3,4,5,6,7,8,9,10,11,12,13]. Weiss and his coworkers developed crosslinked elastin hydrogels for regenerative medicine [4,9,11]. Mithieux *et al.* [4] reported biocompatible hydrogels formed by chemical crosslinking of recombinant human tropoelastion with bis(sulfosuccinimidyl) suberate. Their hydrogels showed good mechanical properties with linearly extension >150%, and supported cellular growth *in vitro* using epithelial cells and fibrosarcoma cells. They also found that their hydrogels was innocuous, compatible, and well-tolerated *in vivo* by implantation in the dorsum of guinea pigs. Some research groups demonstrated that elastin-based materials have great potential to be used for regeneration of damaged cartilage [12,13]. However, isolation of water soluble elastin polypeptides from living bodies or food-processing waste without denaturing is difficult, and it requires some technical procedures to be undertaken because of the crosslinking. *In vitro* synthesis of polypeptides with a sequence derived from elastin and recombinant tropoelastin is also technical and not suitable for mass production due to its cost. On the other hand, fish elastin peptide with a very low molecular weight (*M*_n_ ~500 g/mol) is reported to be isolated by an easy procedure [14], and commercially available at a low cost. Shiratsuchi *et al.* showed that soluble elastin peptide enhanced the proliferation and migration of fibroblast [15]. Therefore, it has great advantages, if it can be used for fabrication of hydrogels with good mechanical properties and extensibility like elastin-containing tissues. In the course of the development of biomaterial technologies, biological materials of marine origin such as marine biopolymers will have the potential to direct the stem cell fate, targeting the delivery of cells and reducing immune rejection, thereby supporting the development of regenerative medicine [16].

**Scheme 1:**
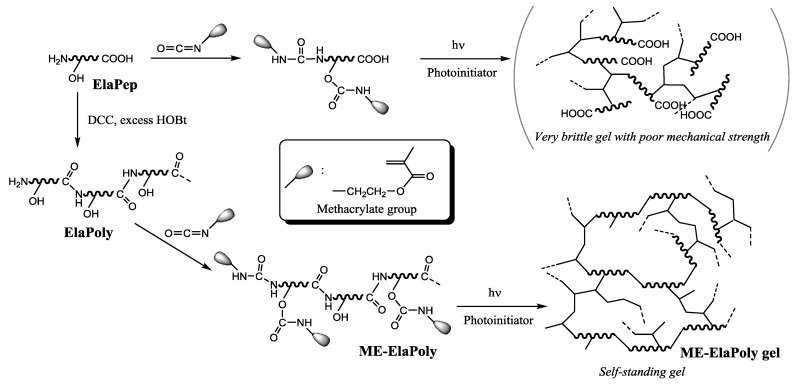
Preparation of photocrosslinked hydrogels from a low-molecular weight elastin peptide through the peptide chain extension prior to the modification with MOI.

At first we prepared a hydrogel by photocrosslinking the low molecular weight elastin peptide with methacrylate groups, which was derived from fish bulbus arteriosus and modified with 2-isocyanatoethyl methacrylate (MOI). However, we obtained a very brittle hydrogel with poor mechanical strength. Therefore, we improved the formulation process and decided to extend the peptide chain by polymerization using a condensation reagent prior to the modification with MOI (Scheme 1). In the polymerization process, our original peptide extension method [17] was applied to control and suppress the undesired crosslinking reaction by adding excess 1-hydroxytriazole (HOBt).

In the present study, microfibrillated cellulose (MFC) was incorporated into the gel for further improvement of the mechanical properties. MFC composed of nano-sized cellulose fibrils can now be obtained from many plant sources and bacteria by several methods; and bleached kraft pulp is most often used for MFC production [18,19,20,21,22,23,24]. MFC is known to improve mechanical properties of materials [23,24,25,26,27,28,29], and its applications to hydrogels have been also reported [27,28,29] as well as other cellulose nanoparticles such as nanocrystals [30,31,32] and whiskers [33,34,35]. Cellulose fibers exhibit a unique structural hierarchy as nanofiber assemblies: A diameter of each nanofiber ranges from 3–5 nm, and some dozens of nanofibers form intertwined aggregates with a width of 20–25 nm in wood [21,23,24,36]. Because cellulose molecules are packed closely and interact with each other in a crystal of a nanofiber, the mechanical strength of the nanofiber is estimated to be very high (at least 2 GPa), although it has not been clarified yet [20,23]. According to the strong mechanical properties of cellulose nanofibers, addition of a small amount of them is expected to improve the mechanical properties of the composite materials. Indeed, many researchers reported the mechanical improvement of polymer materials [23,24,25,26]. As far as the mechanical improvement of hydrogels, Nair *et al.* [27] prepared hydrogels by crosslinking nanofibrillated cellulose with poly(methyl vinyl ether-co-maleic acid) and polyethylene glycol. The mechanical strength and modulus of the composite hydrogels increased with an increase of nanofibrillated cellulose. Borges *et al.* [28] reported the mechanical properties and biocompatibility of composite hydrogels prepared from Tween 20 trimethacrylate, *N*-vinyl-2-pyrrolidone and cellulose nanofibers. The cellulose nanofiber-reinforced hydrogels showed higher elastic modulus than the neat hydrogels. Their hydrogels had no toxic effect on cartilage fetal cells.

In the present study, we successfully prepared self-standing hydrogels from a low-molecular weight fish elastin peptide and improved its mechanical properties using commercially-available microfibrillated cellulose. Furthermore, for their application as biomaterials, the fibroblast proliferation was also tested.

## 2. Results and Discussion

### 2.1. Synthesis of a Methacrylate-Modified Fish Elastin Polypeptide (ME-ElaPoly)

A fish elastin peptide (ElaPep) we used in the present study was extracted from the bulbus arteriosus of skipjacks by protease hydrolysis [14], and its molecular weight was very low (*M*_n_ ~500 g/mol measured by GPC (see Figure S1)). Because elastin is mainly composed of amino acids without a reactive side chain such as Gly, Ala, Pro, and Val, fabrication of self-standing materials from an elastin peptide with a very low molecular weight is not easy. Therefore, we firstly polymerized the peptide with our original peptide extension method using *N*,*N*′-dicyclohexylcarbodiimide (DCC) and excess 1-hydroxybenzotriazole (HOBt) [17], by which the undesired crosslinking reaction was suppressed and a soluble polypeptide was obtained. DCC is a well-known condensation reagent connecting a carboxyl group to an amino group or a hydroxyl group. When a peptide is polymerized by the conventional condensation method, in which a slightly excessive amount of HOBt is added (~1.2 eq.) to carboxyl groups, the product becomes inhomogeneous, insoluble matter by crosslinking, or the polydispersion index (PDI) of the product becomes very high even if it is soluble. We found that a soluble polypeptide with relatively low PDI (~4) was obtained by adding excess HOBt (ElaPep:DCC:HOBt = 1:4:4, molar ratio). The number-average molecular weight (*M*_n_) of the product (ElaPoly) was 6200 g/mol and the weight-average molecular weight (*M*_w_) was 26,000 g/mol (see Figure S2).

**Figure 1:**
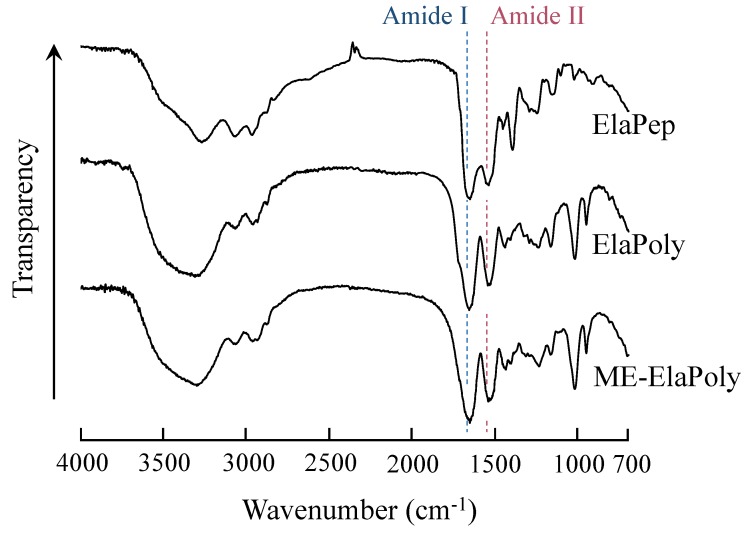
FT-IR spectra of ElaPep, ElaPoly, and ME-ElaPoly.

**Figure 2:**
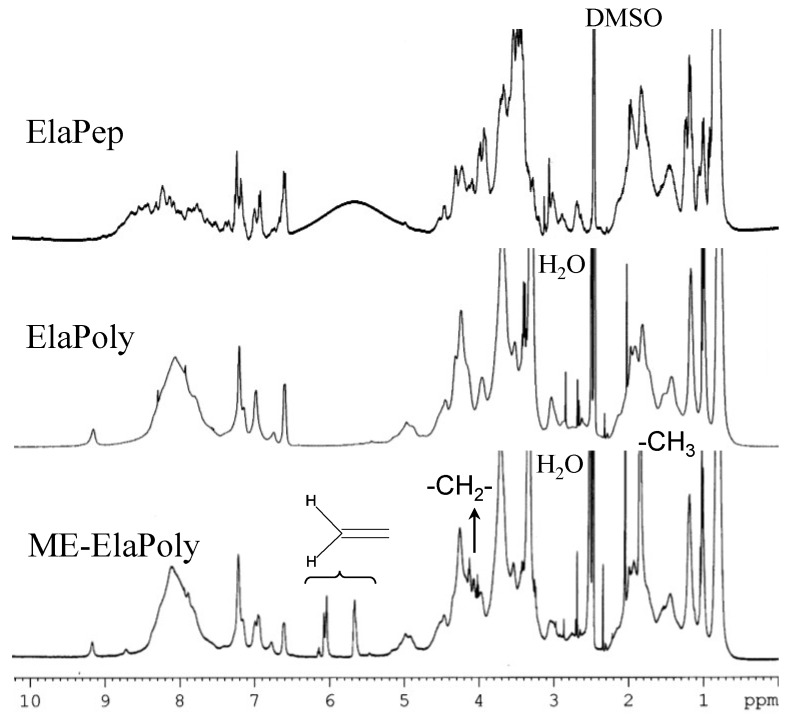
^1^H NMR spectra of ElaPep, ElaPoly, and ME-ElaPoly.

After purification of ElaPoly, the modification reaction with 2-methacryloyloxyethylisocyanate (MOI) was carried out in dimethylsulfoxide (DMSO). The reaction product (ME-ElaPoly) was soluble in DMSO and *N*,*N*-dimethylformamide (DMF) but slightly soluble in water. Since ElaPep and ElaPoly are soluble in DMSO and water but insoluble in DMF, ME-ElaPoly is more hydrophobic than ElaPep and ElaPoly. Figure 1 shows the FT-IR spectra of ElaPep, ElaPoly, and ME-ElaPoly, and Figure 2 shows the ^1^H NMR spectra of ElaPep, ElaPoly, and ME-ElaPoly. It is known that a peptide bond has two absorption bands of amide I at 1680–1620 cm^−1^ and amide II at 1510–1580 cm^−1^ in FT-IR spectroscopy. Though these two absorption peaks did not split clearly in the spectrum of ElaPep, they split clearly at 1650 cm^−1^ (amide I) and 1540 cm^−1^ (amide II) in that of ElaPoly. This result indicates the formation of peptide bonds. After the reaction of ElaPoly with MOI, the increase in the absorbance related with methacrylate groups and urethane bonds was expected. However, the increase was not observed clearly, because the modification with MOI only partially occurred. The modification was confirmed clearly by the NMR spectral changes. In the NMR spectrum of ME-ElaPoly, four signals appeared. Two signals corresponding to the unsaturated methacrylate groups were observed at 6.1 ppm and 5.6 ppm, and a signal corresponding to the methyl protons of the methacrylate group was observed at 1.8 ppm. A signal corresponding to the methylene protons of –O–CH_2_– was observed at 4.1 and 4.2 ppm. A signal corresponding to the methylene protons of –CH_2_–N< was not observed, because the signal overlaps the signal of water at 3.4 ppm. These results confirmed the synthesis of ME-ElaPoly. The isocyanate groups of MOI react with hydroxyl groups, amino groups, mercapto groups, and carboxyl groups. Since the content of amino acids that react with isocyanate groups—that is Asp, Cys, Glu, Hyp, Lys, Ser, Thr, and Tyr—is obtained from the amino acid composition of ElaPep, the degree of conversion can be calculated using signal integral values of the ^1^H NMR spectra. We calculated the degree of conversion, and found that the degree of conversion for ME-ElaPoly was 26%.

### 2.2. Preparation of ME-ElaPoly/MFC Composite Gels by Photocrosslinking

**Figure 3:**
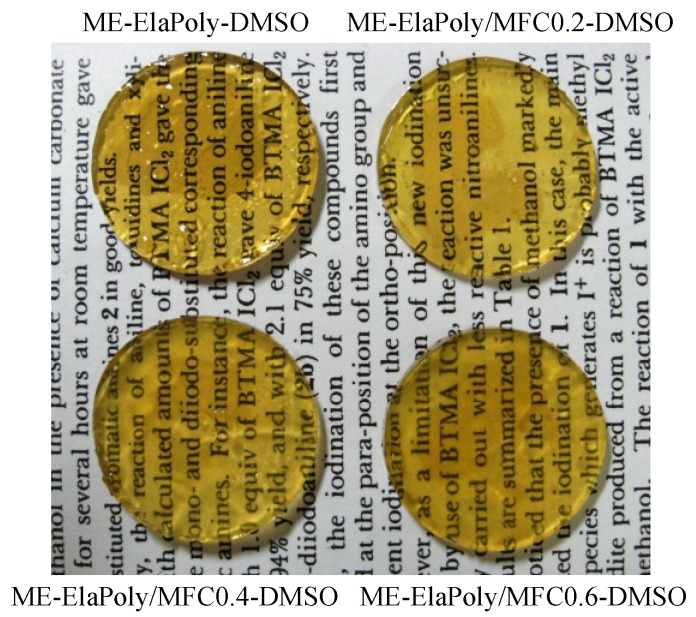
Appearance of ME-ElaPoly-DMSO organogel in the presence and absence of MFC.

**Figure 4:**
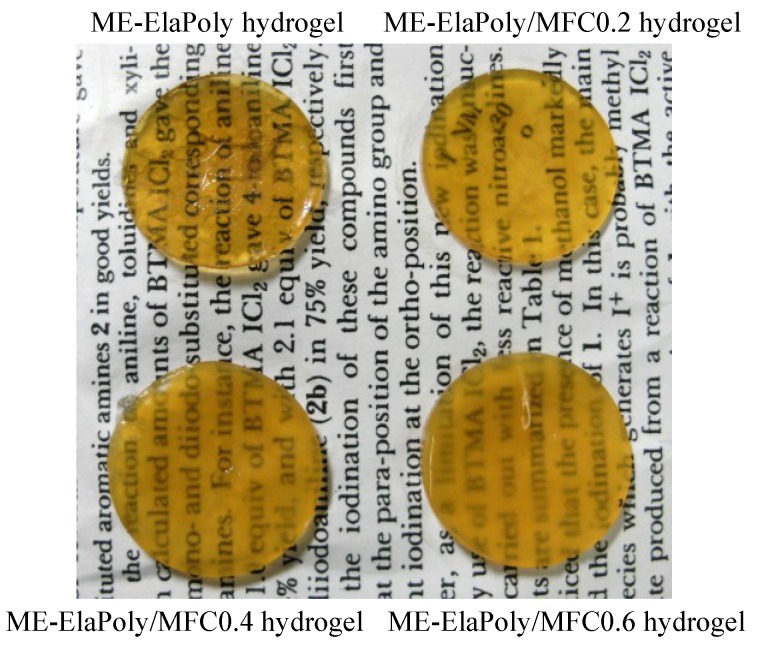
Appearance of ME-ElaPoly hydrogel in the presence and absence of MFC.

Since ME-ElaPoly is soluble in DMSO but insoluble in water, photocrosslinked gel was prepared by photoirradiation of DMSO solution of ME-ElaPoly in the presence of photoinitiator followed by solvent exchange with water. The concentration of MFC was varied from 0.2%–0.6% against the whole weight of gel. The composite gel samples prepared in the present study were designated as “ME-ElaPoly/MFC X”, where X is the concentration of MFC (in weight percentage). ME-ElaPoly-DMSO organogel was a transparent gel with yellowish color. As-prepared ME-ElaPoly/MFC-DMSO organogels were also transparent, and the increase of MFC content did not significantly affect the appearance (Figure 3). After solvent exchange with water, the gels slightly shrunk and became turbid (Figure 4). The turbidity of ME-ElaPoly/MFC hydrogels increased with MFC content.

We consider two possible reasons for the turbidity. First, transparency of composite gel materials depends on the difference of the refractive index (RI) between dispersing particles and solvent. If the difference becomes larger, the composite gel becomes more turbid. RI of DMSO is 1.48, and RI of water is 1.33, while RI of cellulose fiber is known to be 1.54–1.62 [37]. The difference of RI became larger because of the solvent exchange from DMSO to water. Secondly, transparency also depends on dispersibility of MFC. If the dispersibility deteriorates, the composite gel becomes more turbid. As well as water, DMSO is also able to be used for preparing cellulose nanocrystal suspension [38]. MFC in our composite gel materials is surrounded by ME-ElaPoly, a hydrophobic polypeptide which is soluble in DMSO with lower solubility in water. The interaction between ME-ElaPoly and MFC may promote the aggregation of MFC.

**Figure 5:**
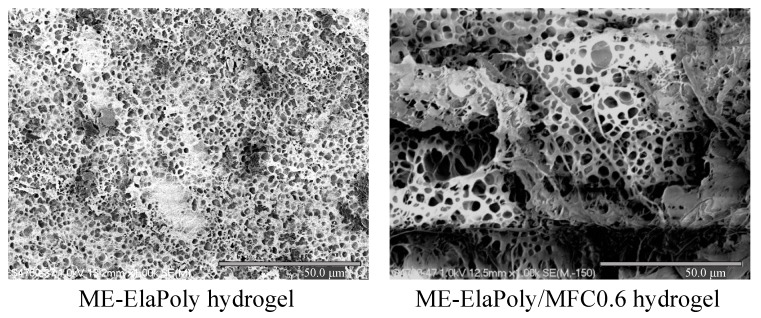
FE-SEM micrographs of the lyophilized ME-ElaPoly and ME-ElaPoly/MFC0.6 hydrogels.

Figure 5 shows morphology of the cross-section of the lyophilized ME-ElaPoly and ME-ElaPoly/MFC0.6 gel observed by FE-SEM. All samples exhibited the porous morphology, and pull-out nanofibers of MFC were observed in ME-ElaPoly/MFC. The pore size was 2~8 μm, and these pores were opened by removal of water from hydrogels. The pore size of ME-ElaPoly/MFC0.6 was larger than that of ME-ElaPoly. MFC embedded in ME-ElaPoly cell walls of the gel was not obviously observed, and some pores were found to align along cellulose fibers. The morphology implies an interaction occurred between ME-ElaPoly and MFC. MFC seemed to disperse homogeneously in ME-ElaPoly networks.

### 2.3. Mechanical Properties of ME-ElaPoly/MFC Composite Gels

**Figure 6:**
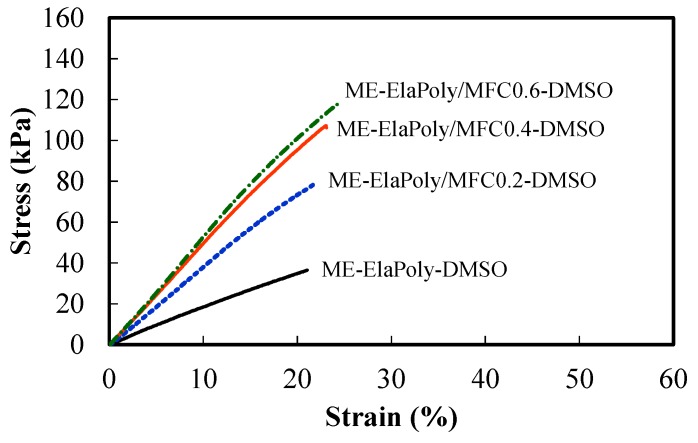
Stress–strain curves of ME-ElaPoly-DMSO organogel, ME-ElaPoly/MFC0.2-DMSO organogel, ME-ElaPoly/MFC0.4-DMSO organogel, and ME-ElaPoly/MFC0.6-DMSO organogel.

**Figure 7:**
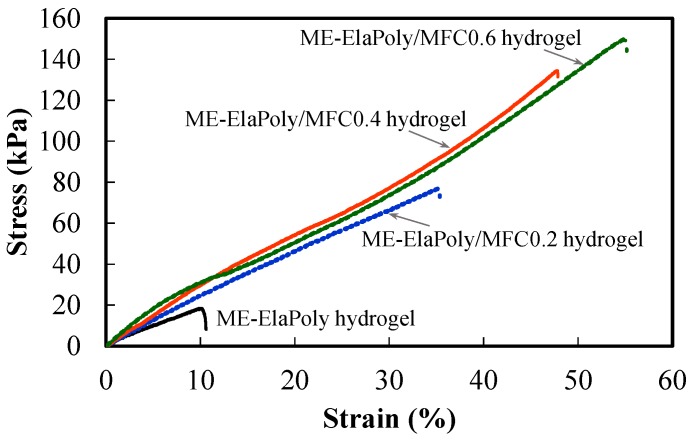
Stress–strain curves of ME-ElaPoly hydrogel, ME-ElaPoly/MFC0.2 hydrogel, ME-ElaPoly/MFC0.4 hydrogel, and ME-ElaPoly/MFC0.6 hydrogel.

The mechanical properties of fish elastin peptide-derived ME-ElaPoly/MFC composite gels were measured by tensile tests. Both the gels swelled with DMSO and those swelled with water were tested. Figure 6 shows the results of stress–strain curves in the tensile tests of ME-ElaPoly/MFC-DMSO organogels. The tensile strength and the modulus of ME-ElaPoly/MFC-DMSO organogels were increased with an increase of MFC content. The strain at break did not significantly change by the addition of MFC. The composite organogels showed the typical behavior of fiber-reinforced materials in which the interaction between matrix and fibers is not strong. On the other hand, the composite hydrogels obtained by the solvent exchange showed interesting behavior in the tensile tests. Figure 7 shows the results of stress–strain curves in the tensile tests of ME-ElaPoly/MFC hydrogels. The tensile strength and the strain at break of ME-ElaPoly/MFC hydrogels were increased with an increase of MFC content. Interestingly, the stress–strain curve of ME-ElaPoly/MFC hydrogels with higher MFC content became characteristic of elastomeric materials. Originally the elastin peptide was obtained from the organs whose mechanical properties are elastic. Though the ME-ElaPoly hydrogel was a brittle gel with very low elasticity, we could partially recover the elasticity of elastin by reinforcing with MFC. Considering that the elasticity was not observed for ME-ElaPoly/MFC-DMSO organogels, we proposed a mechanism for the recovery of the elasticity as follows. ElaPoly was synthesized by polymerization of the elastin peptide and it partially has a secondary structure of elastin. The secondary structure was considered to be deformed in DMSO and restored in water. Our results can be explained by making a hypothesis that the elasticity derived from the secondary structure was supported markedly by MFC because of interaction between ElaPoly and MFC.

Though our composite hydrogels showed somewhat deteriorated mechanical properties compared to the crosslinked hydrogel prepared from recombinant human tropoelastin [4], the mechanical strength of our hydrogels is as strong as that of the interpenetrating polymer network of poly(ethylene glycol) and chitosan [39], and higher than that of photocrosslinked poly(*N*-vinyl-2-pyrrolidone) hydrogels [40] and that of dextran/gelatin hydrogels [41]. Furthermore, ME-ElaPoly is considered to have branched chains, because of some chemical condensation reaction that involves bonding at side chains. Generally, the existence of branches is known to deteriorate the mechanical properties [42,43]. In the present study, we succeeded in the recovery of mechanical properties not only by the chain extension but also by the complexation with MFC. Through our strategy, we could obtain fish elastin peptide-derived hydrogels with highly improved mechanical properties, which endure common handling.

We compared our composite gels to biological tissues on the base of mechanical properties. The tensile strength and the Young’s modulus of ME-ElaPoly/MFC0.6 hydrogel are ~150 kPa and ~350 kPa, respectively. For example, with regards to biological tissues, the tensile strength and the modulus of human Achilles tendon are ~70 MPa and ~800 MPa [44], respectively; those of human lumber anterior longitudinal ligament are ~27 MPa and ~760 MPa [45]; those of human articular cartilage of the femoral condyle are ~10 MPa and ~15 MPa [46]; the strength of human stratum corneum (skin) is ~300 kPa at 100% R.H. [47]; the burst pressure of human saphenous vein is ~200 kPa (~1600 mmHg), and the burst pressure of human internal mammary artery is ~400 kPa (~3200 mmHg) [48]. The mechanical properties of our composite gels are comparable to those of skin, vein, and artery.

**Figure 8:**
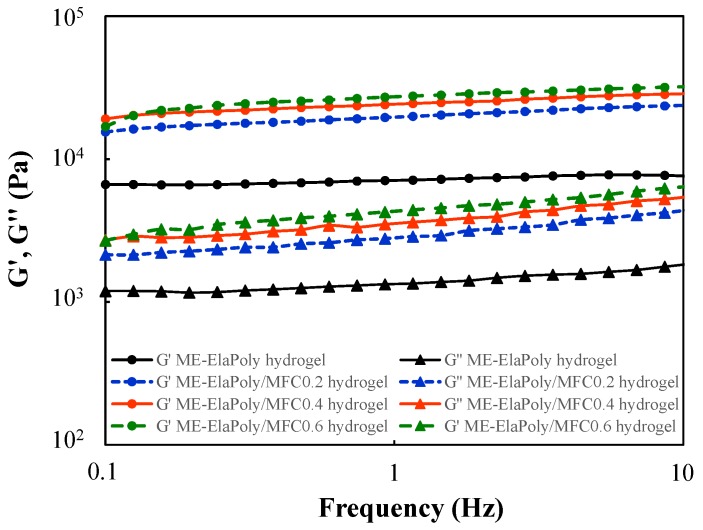
Viscoelastic properties of ME-ElaPoly hydrogel, ME-ElaPoly/MFC0.2 hydrogel, ME-ElaPoly/MFC0.4 hydrogel, and ME-ElaPoly/MFC0.6 hydrogel.

Figure 8 shows the viscoelasticity of ME-ElaPoly/MFC hydrogels in the frequency range of 0.1–10 Hz. All samples showed the typical gel behavior with storage modulus (G′) being higher than loss modulus (G″) over the whole frequency range. G′ was independent of oscillatory frequency, and increased with an increase of MFC content. G″ of ME-ElaPoly/MFC hydrogel increased gradually with an increase of frequency. The slight change in G″ was shown also in the gelatin-derived hydrogel reinforced with imogolite nanofibers [49].

### 2.4. Cell Proliferation Assay

**Figure 9:**
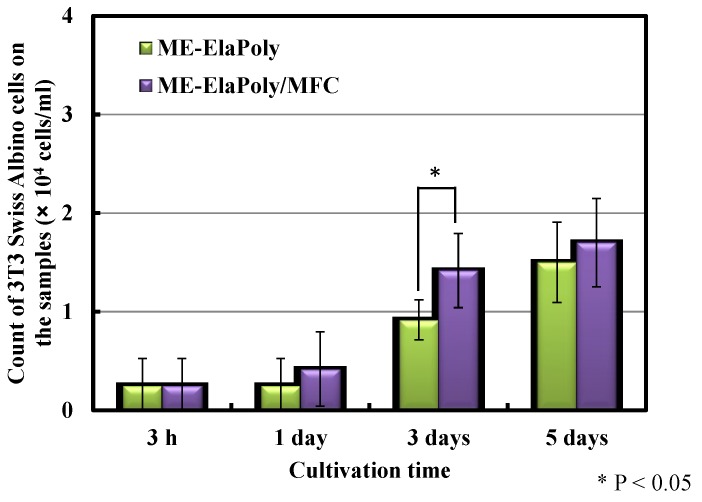
Cell proliferation assay by counting cells using a hemocytometer. Following removal of floating cells, the cells that adhered to each sample were collected after trypsin treatment. The error bars represent standard deviation of mean. Statistical significance was represented by *p* < 0.05 (*).

**Figure 10:**
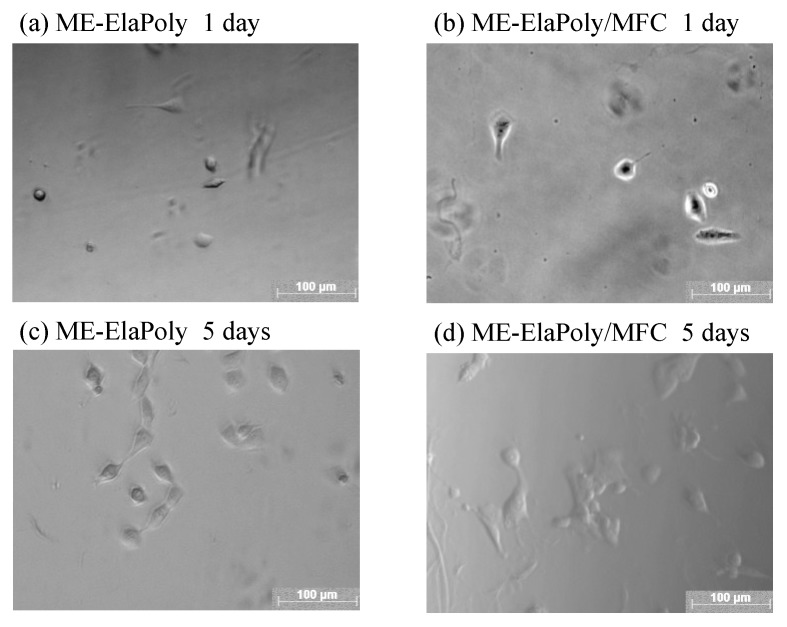
Phase contrast microscope observation of grown cells on (**a**) ME-ElaPoly and (**b**) ME-ElaPoly/MFC after 1-day culture, and cells on (**c**) ME-ElaPoly and (**d**) ME-ElaPoly/MFC after 5-day culture.

Cell proliferation on ME-ElaPoly and ME-ElaPoly/MFC hydrogel was assayed using 3T3 Swiss Albino mouse embryo fibroblast cells. Figure 9 shows the numbers of cells that adhered to each sample. There were very few cells that were dead on ME-ElaPoly and ME-ElaPoly/MFC. The proliferation was not higher than PS culture plate that was optimized for cell culture. The similar results were also observed in a study where vascular smooth muscle cells were seeded on an elastin-based material crosslinked with ethylene glycol diglycidyl ether [10]. After 1-day and 3-day culture, the number of the cells adhered to ME-ElaPoly/MFC was higher than that on ME-ElaPoly. After 7-day culture, however, there was no significant difference between these two materials. The result suggests that the existence of MFC presumably promotes the initial attachment of cells, but does not influence the cell proliferation. Figure 10 shows the morphology of the cells attached onto ME-ElaPoly and ME-ElaPoly/MFC. After 3-day culture, there existed many spread cells on ME-ElaPoly/MFC. After 7-day culture, most cells spread and gathered on both substrates. Fibroblast cells attached to materials are known to secrete cell growth factors such as fibronectin [50]. Therefore, it is reasonable that fibroblast cells gathered.

## 3. Experimental Section

### 3.1. Materials

A fish elastin peptide (ElaPep, the number average molecular weight (*M*_n_) ~500 g/mol measured by GPC) which was extracted from the bulbus arteriosus of skipjacks was supplied by Hayashikane Sangyo Co., Ltd. (Yamaguchi, Japan). 1-Hydroxybenzotriazole (HOBt) and 2-methacryloyloxyethylisocyanate (MOI) were purchased from Tokyo Chemical Industry Co., Ltd. (Tokyo, Japan). *N*,*N*′-Dicyclohexylcarbodiimide (DCC) and anhydrous DMSO were purchased from Sigma-Aldrich Co. (St. Louis, MO, USA). Other organic solvents were purchased from Kanto Chemical Co., Inc. (Tokyo, Japan); and a minimum essential medium alpha modification (MEM-α) and a penicillin-streptomycin mixture (10,000 U/mL penicillin and 10,000 mg/mL streptomycin) were purchased from Wako Pure Chemical Industries, Ltd. (Osaka, Japan). These chemical reagents were used as received. Irgacure 2959, 2-hydroxy-1-[4-(hydroxyethoxy)phenyl]-2-methyl-1-propanone, was kindly provided from Ciba Specialty Chemicals K.K. (Tokyo, Japan) and used as received. Celish KY100G, which contains 10% MFC in water, was kindly provided by Daicel FineChem Ltd. (Tokyo, Japan). Ultrapure water (electric resistance >18 MΩ·cm^−1^) used for gel preparation was obtained through Millipore Direct-Q (Merck Millipore, Billerica, MA, USA) and used for preparation of imogolite and hydrogel.

### 3.2. Polymerization of a Fish Elastin Peptide (ElaPep)

ElaPep (8.05 g) was dissolved in 80 mL of anhydrous DMSO, and HOBt (13.2 g) was added to the solution of ElaPep. After DCC (8.66 g) was added, the solution was stirred at 700 rpm for 24 h at room temperature using an EYELA ChemiStation PPS-5510 personal organic synthesizer (Tokyo Rikakikai Co., Ltd., Tokyo, Japan). After the reaction, the solution was filtered to remove dicyclohexylurea and the filtrate was poured dropwise into excess acetonitrile. The mixture was stirred for 24 h at room temperature, and then filtered with filter paper. The product on the filter paper was dissolved in 40 mL of DMSO, and reprecipitated in acetonitrile. The dispersion was stirred for 24 h at room temperature for washing, and filtered again. To remove the residual volatile impurities, the product was dried in vacuo for 2 days to yield white powder (ElaPoly, yield 70%).

### 3.3. Synthesis of a Methacrylate-Modified Fish Elastin Polypeptide (ME-ElaPoly)

ElaPoly (3.00 g) was dissolved in 30 mL of anhydrous DMSO by stirring for 24 h at room temperature, and MOI (1.03 g) was added dropwise. The solution was stirred at 700 rpm for 24 h at 60 °C using an EYELA ChemiStation PPS-5510 personal organic synthesizer. After the reaction, the solution was poured dropwise into excess acetonitrile and the mixture was stirred for 1 h at room temperature. The mixture was filtered with filter paper, and the product on the filter paper was washed with ethanol. To remove the residual volatile impurities, the product was dried in vacuo for 1 day to yield white powder (ME-ElaPoly, yield 68%).

### 3.4. Preparation of ME-ElaPoly/MFC Composite Gels by Photocrosslinking

Celish KY100G, which contains 10 wt% of MFC and 90 wt% of water, was dispersed in DMSO to exchange the solvent, followed by stirring for 2 h and centrifuging at 3500 rpm for 10 min. After this procedure was repeated twice, the resulting precipitate was dispersed in DMSO and sonicated for 1 min using a Branson Sonifier 250 ultrasonic homogenizer (Branson Ultrasonics Co., Danbury, CT, USA). Then ME-ElaPoly was dissolved in the MFC dispersion in DMSO at the final concentration of 30 wt% in the solution. The concentration of MFC in the gel was varied from 0.2–0.6 wt%. Irgacure 2959, a radical photoinitiator, was added to the mixture at the concentration of 0.2 wt%. The mixture (3 g) was poured into a small Teflon container with an inner diameter of 30 mm and an inner height of 10 mm, and irradiated with UV light (~60 mW/cm^2^) for 4.5 min using a Spotcure SP-7 UV irradiator (Ushio Inc., Tokyo, Japan) equipped with a light guide for homogeneous irradiation. To cut off the light of wavelength shorter than 300 nm, the irradiation was carried out through a glass plate of 1 mm thickness. The samples for the tensile test were prepared using a 1-mm thick silicone plate with rectangle holes of 5-mm width and 40-mm length as a mold, which is sandwiched between two glass plates. The as-prepared gels containing DMSO (ME-ElaPoly/MFC-DMSO organogels) were immersed in excess ultrapure water for 24 h at room temperature in order to exchange the solvent, yielding ME-ElaPoly/MFC hydrogels.

### 3.5. Characterization

The surface morphology of lyophilized gel samples was observed by a Hitachi S-4700 field emission scanning electron microscope (FE-SEM) (Hitachi High-Technologies, Tokyo, Japan). The samples were coated with gold prior to the observation, and the accelerating voltage was 1 kV.

The tensile test was carried out by a Shimadzu EZ-S tabletop universal tester (Shimadzu Corp., Kyoto, Japan) with a 100 N load cell at a crosshead speed of 1 mm/min using rectangle specimens with a width of 5 mm and a length of 40 mm. In each case, six specimens were tested and the average values were calculated. The dynamic viscoelasticity of the hydrogel was measured by a DAR-100 rheometer (Rheologica Instruments AB, Lund, Sweden) equipped with a plate geometry with a diameter of 20 mm. The storage and loss moduli (G′ and G″) were determined as a function of frequency under the condition of a linear viscoelastic response at 25 °C.

### 3.6. Cell Proliferation Assay

Round-shape cover glasses (15 mm diameter) coated by the dip coating method using an ME-ElaPoly solution in DMSO with dispersed MFC were placed in a 24-well polystyrene (PS) culture plate, and UV light was irradiated using a Spotcure SP-7 UV irradiator. The gel films on the cover glasses were washed with sterilized water several times and then sterilized with 10 kGy γ-ray irradiation. After washing with Dulbecco’s modified Eagle’s medium (D-MEM), 3T3 Swiss Albino mouse embryo fibroblast cells were seeded at the 3 × 10^3^ cells/mL. The cells were incubated at 37 °C in D-MEM containing 10% FBS and 1% penicillin-streptomycin mixture using a CO_2_ incubator (Astec Co. Ltd., Fukuoka, Japan) with 5% CO_2_. Cell growth was observed using a Carl-Zeiss Axio Vert.A1 phase-contrast microscope (Carl-Zeiss AG, Oberkochen, Germany). The number of the cells adhered onto each sample was determined using a hemocytometer (AS ONE Corp., Osaka, Japan) after treated with 0.25% trypsin-EDTA solution and trypan blue staining. The number of replicate wells was 5 (*n* = 5), and differences in proliferation of cells were examined using Student’s *t*-test. Results were deemed statistically significant when *p* < 0.05.

## 4. Conclusions

We successfully prepared photocrosslinked fish elastin polypeptide/MFC composite gels by the chemical extension reaction of short elastin peptide and modification with methacrylate groups, followed by photocrosslinking in the presence of MFC. The solvent substitution of gels from DMSO with water changed the mechanical properties of the composite gels. After the solvent substitution, he composite gels showed a characteristic stress–strain curve with higher strain at break compared with the gel without MFC. The results suggest that some interaction occurred between fish elastin polypeptide and MFC, and the interaction restored the instinct elastic properties of elastin. The photocrosslinked gels supported the growth of fibroblast cells, and showed no toxicity. In the present study, we emphasize the development of a new protocol to change short elastin peptide into self-standing gel materials with flexibility. Our composite hydrogels showed mechanical strength as high as interpenetrating polymer network of poly(ethylene glycol) and chitosan, and higher mechanical strength than photocrosslinked poly(*N*-vinyl-2-pyrrolidone) hydrogels and dextran/gelatin hydrogels. In future study, we will investigate the cell growth using other cell lines such as chondrocytes, osteoblasts, and vascular smooth muscle cells for regenerative medicine. The future study will elucidate the application potential more clearly. Since elastin polypeptides are expected to be degraded gradually in mammalian body, hydrogels derived from elastin polypeptide have an advantage as implantable biomaterials. In addition, considering that elastin is observed in many tissues in our body, a wide range of applications of composite gels is expected.

## Supplementary Files

Supplementary File 1
