# Supplementary material for: Preparation of Photocrosslinked Fish Elastin Polypeptide/Microfibrillated Cellulose Composite Gels with Elastic Properties for Biomaterial Applications
Source: Mar Drugs. 2015 Jan 9;13(1):338–53. doi: 10.3390/md13010338 (PMC4306940; doi:10.3390/md13010338)
Supplement: Supplementary File 1 [file marinedrugs-13-00338-s001.pdf]

# Supplementary Information

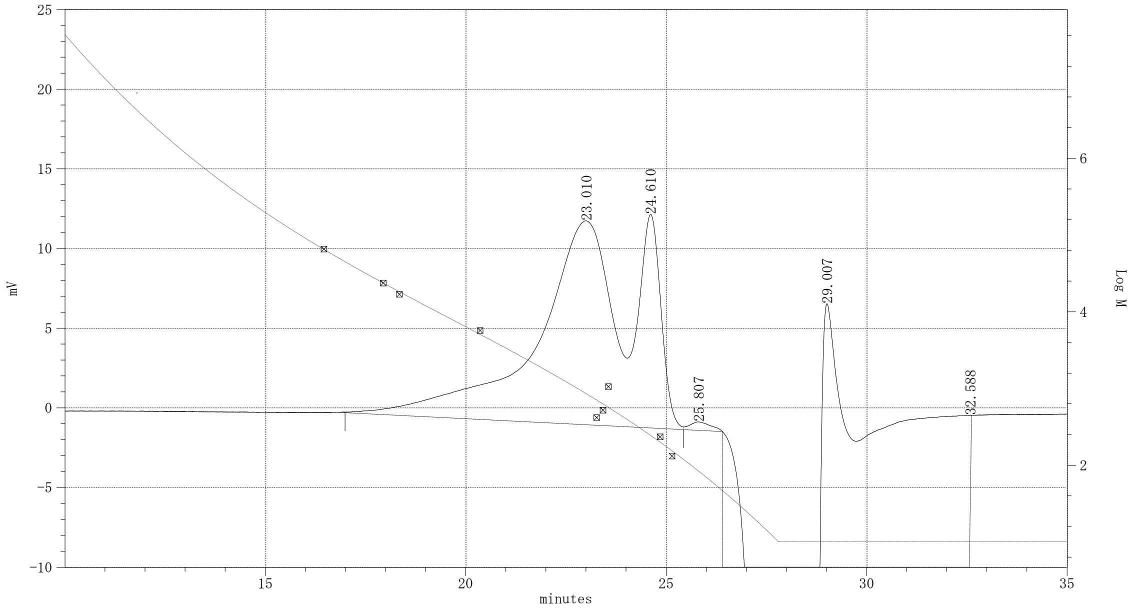


**Figure S1.** GPC profile of ElaPep supplied by Hayashikane Sangyo Co., Ltd. ElaPep was obtained by enzymatic extraction from the bulbus arteriosus of skipjacks. $M_n$ = 500, $M_w$ = 1900.

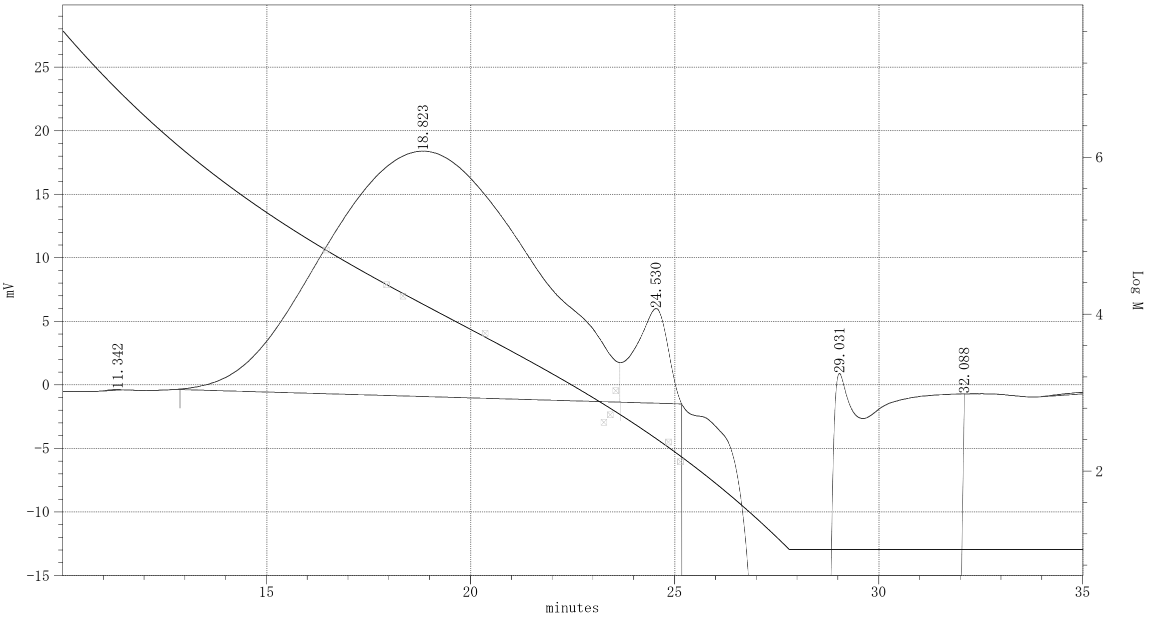


**Figure S2.** GPC profile of ElaPoly (the product of the polymerization of ElaPep). $M_n$ = 6200, $M_w$ = 26,000.

© 2015 by the authors; licensee MDPI, Basel, Switzerland. This article is an open access article distributed under the terms and conditions of the Creative Commons Attribution license (http://creativecommons.org/licenses/by/4.0/).
